# Immunomodulatory Effects of *Pelargonium sidoides* Extract (EPs7630) in the Treatment of Acute Rhinosinusitis

**DOI:** 10.1017/erm.2025.10013

**Published:** 2025-07-15

**Authors:** Aleksandar Perić, Sandra Vezmar Kovačević, Aleksandra Barać, Aneta Perić, Danilo Vojvodić

**Affiliations:** 1 Department of Otorhinolaryngology, Faculty of Medicine of the Military Medical Academy, University of Defense, Belgrade, Serbia; 2 Department of Pharmacokinetics and Clinical Pharmacy, University of Belgrade Faculty of Pharmacy, Belgrade, Serbia; 3 Clinic of Infectious and Tropical Diseases, Clinical Center of Serbia, University of Belgrade Faculty of Medicine, Belgrade, Serbia; 4 Institute of Pharmacy, Faculty of Medicine of the Military Medical Academy, University of Defense, Belgrade, Serbia; 5 Institute of Medical Research, Division of Clinical and Experimental Immunology, Faculty of Medicine of the Military Medical Academy, University of Defense, Belgrade, Serbia

**Keywords:** bacteria, chemokines, cytokines, inflammation, nasal mucosa, pelargonium, polyphenols, sinusitis, viruses

## Abstract

**Background:**

In this short narrative review, we would like to discuss the immunomodulatory effects of South African geranium (*Pelargonium sidoides*) root extract EPs7630 in treating acute rhinosinusitis. The plant has been used for centuries to treat respiratory tract inflammation, such as sinusitis, pharyngitis and bronchitis. South African geranium is rich in polyphenols, flavonoids, tannins, diterpenes and proanthocyanidins, but the main constituent is a type of coumarin called ‘umckalin’ (6–hydroxy–5,5–dimethoxy–coumarin). The substance is standardised as an aqueous-ethanolic extract from the root of this plant under the code name EPs7630.

**Methods:**

The article presents the results of *in vitro* and *in vivo* studies of administering this herbal drug in acute viral, post-viral and bacterial rhinosinusitis. The focus is on the immunomodulatory effects of EPs7630 during the therapy of this acute inflammation of the nasal mucosa.

**Results:**

According to the results of some studies, EPs7630 stimulates monocyte-dependent activity and inhibits neutrophil-dependent chemokine activity. However, given the small number of studies, the level of evidence is low, implying the need for new research.

**Conclusion:**

Particular attention should be paid to the effect of EPs7630 on bradykinin, the mediator that triggers most inflammatory processes in acute rhinosinusitis.

## Introduction

Acute rhinosinusitis (ARS) is a heterogeneous clinical entity in terms of aetiology, pathogenesis and severity of symptoms and signs. According to the EPOS 2020 guideline for diagnosis and therapy of rhinosinusitis, ARS lasts up to 12 weeks (Ref. 1). Diagnosis is based on medical history and physical examination, including rhinoscopy and nasal endoscopy (Refs. 1–4). Factors predisposing to the development of ARS include allergic rhinitis, anatomical variations in the lateral nasal wall that impair sinus ventilation and drainage, ciliary dyskinesia, air pollution and active and passive smoking (Refs. 1–8). ARS occurs primarily as a viral infection of the nasal mucosal layer in over 98% of cases (Refs. 1, 2, 9–11). Rhinoviruses cause inflammation in about 50% of viral infections, and their binding to epithelial cells of the nasal mucosa is favoured by the release of intercellular adhesion molecule 1 (ICAM-1) (Refs. 1, 2, 7–9). Other viral pathogens are coronaviruses, including severe acute respiratory syndrome coronavirus 2 (SARS-CoV-2), adenoviruses, respiratory syncytial viruses, influenza and parainfluenza (Refs. 1, 2, 9, 10). During inflammation, viruses trigger a strong immune response driven by various pro-inflammatory cytokines and chemokines and bradykinin, a potent inflammatory mediator that has a very important role in the pathogenesis of bacterial infection and acute inflammation (Refs. 9–11). The symptoms of ARS can be divided into ‘systemic’ and ‘local’ symptoms. Systemic symptoms, such as fever, muscle aches, headache and malaise, are the result of the release of cytokines and chemokines from neutrophils and lymphocytes (Ref. 12). Bradykinin mainly causes local symptoms, such as nasal congestion, runny nose, sinus pain and sneezing due to stimulation of the sensory endings of the trigeminal nerve (Ref. 12). The weakened sense of smell is a consequence of the combined effect of bradykinin and proinflammatory cytokines on the olfactory neuroepithelium, which is particularly pronounced in the influenza virus and SARS-CoV-2 infection (Ref. 12). Symptoms, such as nasal obstruction, increased nasal secretions, postnasal discharge, pain and pressure in the face and forehead and a weakened sense of smell, subside within 10 days (Refs. 1, 2, 11). However, in 17–21% of cases, the inflammatory process in the mucosa persists even without the presence of a virus, leading to acute post-viral rhinosinusitis (APRS) with the prolongation and worsening of symptoms and signs for up to 12 weeks (Refs. 1, 4, 5, 11). In only 0.5–2% of cases, ARS occurs as a primary bacterial inflammation, acute bacterial rhinosinusitis (ABRS) (Refs. 1, 4, 5, 11). The symptoms worsen after the fifth day: the nasal secretions become purulent, the pain in the projection of the sinuses increases and the body temperature remains above 38.5 degrees, with elevated levels of C-reactive protein (Refs. 1, 4, 5, 11).

The fact that the vast majority of patients with ARS suffered from a viral infection points to the unreasonable use of antibiotics in the treatment of this disease. This was particularly pronounced in certain parts of the world during the coronavirus disease 19 (COVID-19) pandemic. The increase in gastrointestinal symptoms, allergic reactions and, above all, the resistance of bacterial strains to a wide range of antibiotics has prompted experts to reconsider the use of other drugs that can effectively eliminate the symptoms of ARS. Part of those medicinal products that could serve as an alternative are herbal medicines (Refs. 1–3). Some of them were the subject of preclinical and clinical studies, and the results recommend them to be a part of official guidelines for the treatment of ARS (Refs. 1, 2).

### *Pelargonium sidoides* root extract (EPs7630)

**Figure 1.**
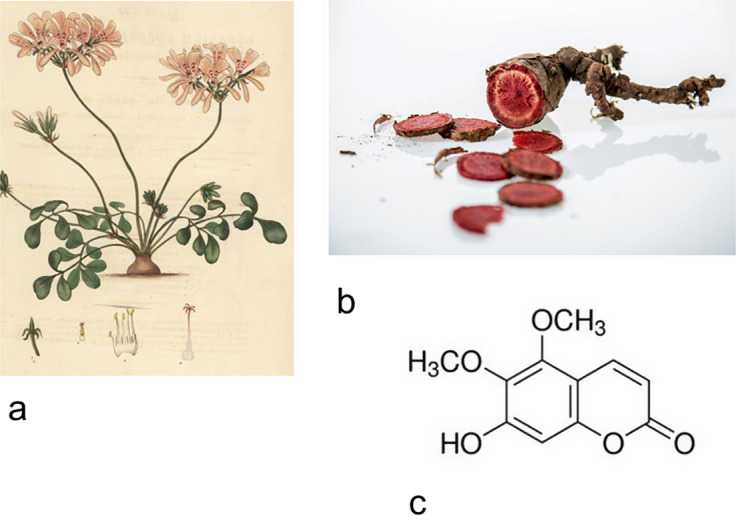
**A**. Appearance of *Pelargonium sidoides* plant; **B.** Appearance of *Pelargonium sidoides* root; **C.** Chemical structure of umckalin.

Root extracts of South African geranium (*Pelargonium sidoides*) have been used for centuries, especially by the indigenous people of South Africa, to treat respiratory and digestive tract infections, such as sinusitis, pharyngitis, bronchitis, tuberculosis, gastroenteritis and others (Refs. 13–15). The plant is rich in polyphenols, flavonoids, tannins, diterpenes and proanthocyanidins, but the main constituent is a type of coumarin called ‘umckalin’ (6–hydroxy–5,5–dimethoxy–coumarin) (Refs. 13–15) (Figure 1). After the plant was brought to Great Britain at the end of the 19th century, a root extract of this plant has been produced in Germany as a standardised drug under the name ‘Umckaloabo’ since the sixties of the 20th century (Refs. 13–15). The drug is standardised as an aqueous-ethanolic extract from the root of this plant under the code name EPs7630 (Refs. 13–15). The drug has been shown to have significant activity against multidrug-resistant strains of *Staphylococcus aureus, Streptococcus pneumoniae, Haemophilus influenzae, Moraxella catarrhalis* and *Streptococcus pyogenes* isolated from the pharynx of patients, with minimal inhibitory concentrations (MICs) > 800 μg/ml for most of the mentioned bacteria (Refs. 14, 15). It has also shown efficacy against influenza type A, respiratory syncytial viruses, coronaviruses, parainfluenza and Coxsackie viruses in inhibitory concentration (IC) values > 100 μg/ml (Refs. 14, 15). This antiviral effect is based on inhibiting the enzyme neuraminidase, which is important for viral replication (Refs. 14, 15). Pharmacological tests have shown its impact on elements of innate and acquired immunity. It stimulates mucociliary transport and has an anti-adhesive effect on bacteria during the infectious phase of the respiratory tract (Refs. 14, 15). This effect was shown to be dose-dependent, and at a concentration of 30 μg/ml, EPs7630 increased the frequency of cilia firing in cultured nasal epithelial cells by 125% (Refs. 14, 15). At the same dose of 30 μg/ml, it significantly increased the phagocytic activity of macrophages and natural killer (NK) cell cultures from the nasal mucosa and stimulated nitric oxide (NO) production (Refs. 14–16). At the concentration of 25 μg/ml, EPs7630 stimulated the production of tumour necrosis factor-α (TNF-α), interleukin 1β (IL-1β) and IL-12 in macrophages cultured from the nasal mucosa (Refs. 14–16). This finding suggests that this herbal drug may increase the resistance of the nasal mucosa to viruses and bacteria (Refs. 14–16).

#### EPs7630 and AVRS

EPs7630 not only blocks the enzyme neuraminidase, which is necessary for the virus to enter the cell and multiply, but also may trigger a strong immune response that works differently from viral infections. The immunomodulatory effect of EPs7630 in viral infections has been demonstrated in three *in vitro* studies. In a study by Witte et al. (Ref. 17), human peripheral blood mononuclear cells (PBMCs) previously infected with the influenza virus and cytomegalovirus (CMV) were treated with EPs7630. The results showed that EPs7630 strongly stimulated the production of the proinflammatory cytokines IL-6 and TNF-α in PBMCs (Ref. 17) (Table 1). This stimulative effect was shown to be dose-dependent, and the first effect on the concentrations of all three cytokines was already visible at a drug concentration of 1 μg/ml. In addition, a less pronounced effect on the anti-inflammatory cytokine IL-10 was observed (Ref. 17). The results suggested the presence of an EPs7630-induced different inflammatory mediator profile from that induced by viral infection, which causes the production of more anti-inflammatory cytokines (Ref. 17) (Table 1). These results suggest that EPs7630 may act as an immunostimulant before viral infection. It could promote innate immune defence and the body’s ability to eliminate potentially invading viruses (Ref. 17) (Table 1). In another *in vitro* study, Witte et al. (Ref. 18) showed that the administration of EPs7630 to a culture of human CD4+ memory T cells and monocytes selectively stimulated the production of IL-17 and IL-22 in these cells at a drug concentration of 3 μg/ml (Table 1). In addition, IL-22 significantly increased the expression of the antimicrobial protective protein S100A9 in the respiratory epithelium. EPs7630 has a strong inhibitory effect on interferon-gamma production (IFN-γ). Thus, it may prevent local mucosal damage by this proinflammatory T1 cytokine (Ref. 18). These results suggest that EPs7630 could replace antibiotics in treating a potential bacterial superinfection in viral sinusitis and bronchitis (Ref. 18) (Table 1).

**Table 1.** Immunomodulatory effects of EPs7630 in the treatment of acute rhinosinusitis

Author	Diagnosis	Type of the study	Effects	References
Witte et al.	AVRS	*In vitro*	EPs7630 dose-dependently induced the production of the proinflammatory cytokines TNF-α and IL–6 in peripheral blood mononuclear cells (PBMCs). These results suggest that EPs7630 may act as an immunostimulant before viral infection. It could promote innate immune defence and the body’s ability to eliminate potentially invading viruses.	(Ref. 17)
Witte et al.	AVRS	*In vitro*	Administration of EPs7630 to the culture of human CD4+ memory T-cells and monocytes selectively enhanced T17 and T22 immune responses by increasing the production of IL–17 and IL–22 in these cells. In addition, IL–22 significantly increased the expression of the antimicrobial protective protein S100A9 in the respiratory epithelium. EPs7630 has a strong inhibitory effect on IFN-γ production and thus prevents local mucosal damage.	(Ref. 18)
Papies et al.	AVRS	*In vitro*	The administration of EPs7630 reduced the ability of SARS-CoV–2 to invade cultured lung epithelial cells by altering the protein composition of the viral spike. In addition, the concentrations of IL–6 and IL–1β were increased, while the levels of IL–8, IL–13, TNF-α, MIG and IP–10 were decreased in the epithelial cell culture fluid.	(Ref. 21)
Perić et al.	APRS	*In vivo* case-control study	After 10 days of oral administration of the herbal medicine EPs7630 (three times daily, 20 mg in tablet form), there is an increase in the concentrations of chemokines associated with monocytes (MCP–1, IP–10 and MIP–1β), and a decrease in the concentration of chemokines associated with neutrophil function (IL–8, GROα and ENA–78). At the same time, there is an improvement in all symptoms and endoscopic findings in APRS.	(Ref. 22)
Perić et al.	ABRS	*In vivo* randomized, prospective, open-label study	After a 10-day administration of EPs7630 (three times daily, 20 mg in tablet form) an improvement in symptoms and endoscopic findings was observed, although the clinical effect of roxithromycin in tablet form (2 x 150 mg/day) was better. In the control group of non-treated persons with ABRS, no improvement occurred after 10 days. In the nasal secretions of patients who did not receive treatment, an increase in the concentration of almost all chemokines was observed after 10 days. Treatment with EPs7630 stimulated the production of MCP–1, MIP–1β and IP–10 and inhibited the production of MIP–1α, ENA–78, GROα and IL–8 in the nasal and paranasal sinus mucosa. Roxithromycin therapy significantly increased the concentration of IP–10 and decreased the concentration of MCP–1, MIP–1α, ENA–78 and IL–8 in the nasal secretions.	(Ref. 27)

The site of entry of SARS-CoV-2 into the human body is, in most cases, the olfactory neuroepithelium (Ref. 19). Although inflammation often has the characteristics of AVRS, it also has its peculiarities, especially the more frequent impairment of the sense of smell and taste, which can affect the patient’s emotional state. Research has shown that olfactory impairment in COVID-19 is due to damage to the sustentacular supporting cells of the olfactory neuroepithelium (Ref. 19). COVID-19 infection harms the speed of mucociliary transport, making the airway mucosa more susceptible to bacterial infection in the post-viral period (Ref. 20). A subsequent *in vitro* study showed that the administration of EPs7630 at a concentration of 10 μg/ml reduced the ability of SARS-CoV-2 to invade cultured lung epithelial cells by altering the protein composition of the viral spike (Ref. 21) (Table 1). In addition, the concentration of IL-6 and IL-1β was increased, while the concentrations of IL-8, IL-13, TNF-α, IFN-γ-induced monokine (MIG) and interferon γ-induced protein 10 kDa (IP-10) were decreased in the epithelial cell culture fluid (Ref. 21) (Table 1). The presence of similar respiratory mucosa in the nose and sinuses could imply similar results related to AVRS. Part of the results related to the production of TNF-α is in contradiction with the previous results of *in vitro* studies, where the stimulatory effect of this extract on the production of this cytokine was reported as strong (Refs. 13–17). This underlines the fact that the results of *in vitro* research depend on which cell cultures are used and on the local conditions prevailing in the laboratory, implying the need for *in vivo* studies.

#### EPs7630 and APRS

**Figure 2.**
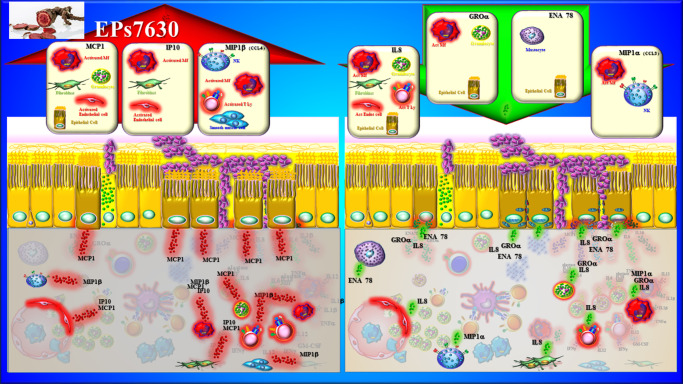
Immunomodulatory effects of EPs7630 in the treatment of APRS. Abbreviations: MCP1: monocyte chemoattractant protein 1; IP10: interferon γ-induced protein 10 kDa; MIP1β: macrophage inflammatory protein 1 beta; IL8: interleukin 8; ENA78: epithelial-derived neutrophil-activating peptide 78; GROα: growth-regulated oncogene alpha; MIP1α: macrophage inflammatory protein alpha; T Ly: T lymphocyte; MF: macrophage; Act. Endot. Cell: activated endothelial cell; Mastocyte: mast cell; NK: natural killer cell.

The pathophysiology of APRS is not entirely clear. In this clinical entity, viral infections trigger numerous changes in the structure of the airway mucosa, including increased infiltration by neutrophils and monocytes and disturbances in host immune response and adaptive immunity (Refs. 1, 3). Infection of the respiratory epithelium by viruses induces strong pro-inflammatory cytokine production. Those cytokines are IL-6, TNF-α, IL-1β, IFN-β and IFN-γ, and the chemokines are IP-10, IL-8 and interferon-inducible T-cell alpha chemoattractant [I-TAC]) (Refs. 1, 3). This increased local production of inflammatory mediators, together with protective surfactant proteins and increased mucus production, is thought to prevent bacterial superinfection but leads to persistent inflammation in the nasal and paranasal sinus mucosa (Refs. 1, 3). Bacteria do not usually play a role in the pathogenesis of APRS. The concentrations of inflammatory mediators in nasal secretions reliably reflect the condition of the nasal mucosa. A previous *in vivo* case–control study has shown that, the concentrations of non-selective chemokines (monocyte chemoattractant protein 1 [MCP-1], macrophage inflammatory protein 1 alpha [MIP-1α], MIP-1β, MIP-3α), which attract various inflammatory cells (monocytes, eosinophils, neutrophils) to the site of acute inflammation, are increased in patients with APRS (Ref. 22) (Table 1). Also, the concentrations of chemokines responsible for attracting and activating neutrophils (IL-8 and epithelial-derived neutrophil-activating peptide 78 [ENA-78]) were locally elevated compared to healthy individuals (Ref. 22). However, after 10 days of oral administration of EPs7630 (three times daily, 20 mg in tablet form), there was an increase in the concentrations of chemokines related to monocytes (MCP-1, IP-10 and MIP-1β) and a decrease in the concentration of chemokines related to neutrophil function (IL-8, growth-regulated oncogene alpha [GROα], ENA-78 and MIP-1α) (Ref. 22) (Figure 2) (Table 1). At the same time, an improvement was shown in all endoscopic findings and signs of APRS (Ref. 22) (Table 1). Thus, as in a viral infection, EPs7630 may stimulate monocyte activity and partially suppress neutrophil activity at the site of acute inflammation. Although this study was not placebo-controlled, these results suggest that EPs7630 could be considered one of the drugs in APRS therapy.

#### EPs7630 and uncomplicated ABRS

Previous studies suggest that EPs7630 may be effective in the treatment of uncomplicated ABRS (Refs. 23–26). A prospective, randomised, open-label study has shown that 10-day use of EPs7630 (20 mg three times daily in tablet form) significantly reduced the incidence of patients with positive cultures of *Streptococcus pneumoniae*, *Haemophilus influenzae* and *Moraxella catarrhalis* from the middle nasal meatus (Ref. 25). In contrast, amoxicillin tablets (3 × 500 mg/day) only reduced the growth of *Streptococcus pneumoniae* and *Haemophilus influenzae* cultures (Ref. 25). The results of the same study showed higher absolute improvement in the total score of nasal symptoms as well as separate nasal symptoms, such as nasal congestion, weakened sense of smell and sense of facial pain and pressure (Ref. 25). In endoscopic findings, patients using EPs7630 had less mucosal oedema and mucopurulent secretions than those treated with amoxicillin (Ref. 25). The explanation for such effects could be the fact that EPs7630 was shown to increase the release of antibacterial peptides (defensins, lactoferrin and bactericidal/permeability-increasing protein [BP-IP]) from neutrophils and increase the phagocytic activity of macrophages against bacteria (Ref. 26).

**Figure 3.**
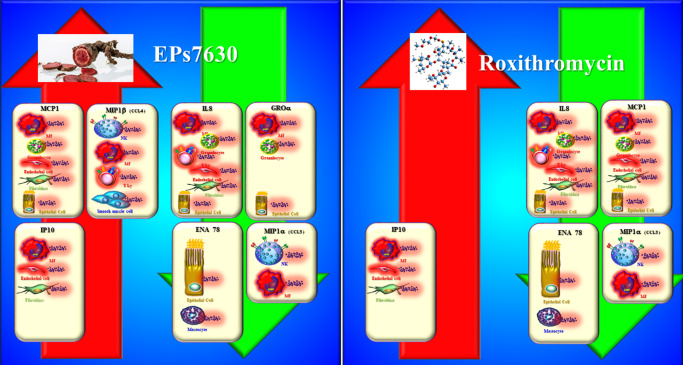
Immunomodulatory effects of EPs7630 and roxithromycin in therapy of uncomplicated ABRS. MCP1: monocyte chemoattractant protein 1; IP10: interferon γ-induced protein 10 kDa; MIP1β: macrophage inflammatory protein 1 beta; IL8: interleukin 8; ENA78: epithelial-derived neutrophil-activating peptide 78; GROα: growth-regulated oncogene alpha; MIP1α: macrophage inflammatory protein alpha; T Ly: T lymphocyte; MF: macrophage; Mastocyte: mast cell; NK: natural killer cell.

In another *in vivo* randomised, prospective, open-label study, the clinical and immunomodulatory effects of the macrolide antibiotic roxithromycin and EPs7630 were compared in the treatment of uncomplicated ABRS (Ref. 27). After a 10-day administration of EPs7630 (three times daily, 20 mg in tablet form), an improvement in endoscopic findings and nasal symptoms was observed, although the clinical effect of roxithromycin in tablet form (2 × 150 mg/day) was better. In the control group of untreated patients with ABRS, there was no improvement after 10 days. This indicates that we cannot expect spontaneous improvement of symptoms and clinical findings in patients with uncomplicated ABRS (Ref. 27) (Table 1). Therefore, medical treatment of ABRS is necessary. In the nasal secretions of patients who did not receive therapy, an increase in the concentration of almost all chemokines was observed after 10 days. Interestingly, similar to APRS, following treatment with EPs7630, the results indicated increased concentrations of MCP-1, IP-10 and MIP-β and decreased levels of MIP-1α, ENA-78, GROα and IL-8 in the nasal secretions (Ref. 27) (Figure 3) (Table 1). Roxithromycin therapy significantly increased the concentration of IP-10 and decreased the concentration of IL-8, ENA-78, MCP-1 and MIP-1α in nasal fluid (Figure 3) (Table 1). The results showed that the two drugs similarly affect the production of chemokines that regulate the function of monocytes and neutrophils in the nasal and paranasal sinus mucosa (Ref. 27).

### Expert summary and future directions

The studies have shown that cytokines and chemokines play an important role in the pathogenesis of all three clinical phenotypes of ARS (Refs. 1, 2, 12). While the role of bradykinin in the pathogenesis of AVRS and ABRS is well documented (Refs. 1, 2, 12), the role of this potent mediator in the pathophysiology of APRS is unclear and needs to be investigated in the near future. Although only five studies explored the immunomodulatory properties of EPs7630, they all showed that administration of the drug stimulated monocyte-dependent activity and inhibited neutrophil-dependent chemokine activity in all three forms of ARS (Refs. 17, 18, 21, 22, 26). However, the results of three studies on antiviral effects are based on laboratory analysis, and it is necessary to have *in vivo* studies. Moreover, the two studies on immunomodulation in the treatment of APRS and ABRS are not sufficient to draw major conclusions. Although the level of evidence is low, the results of the studies may suggest that the extract of *Pelargonium sidoides* could be an option in the therapy of AVRS and APRS and could replace or reduce the use of antibiotics in the treatment of uncomplicated ABRS. Particular attention should be paid to the use of plant extracts concerning their effect on bradykinin, the mediator that triggers most inflammatory processes in ARS. Recent research has shown that the cytokine storm in COVID-19 is triggered by bradykinin, so blocking bradykinin receptors could reduce its effects (Refs. 28, 29). The results of an experimental study in mice, in which the application of gel from the leaves of *Ipomoea* (*Convolvulaceae*) on skin oedema by blocking bradykinin activity has an anti-inflammatory, anti-oedematous and wound-healing effect, are encouraging (Ref. 30). This is where research in the field of phytotherapy should start when it comes to inflammation of the mucous membranes of the upper respiratory tract.

## Data Availability

All data obtained or analysed as part of the study are included in this published article.

## References

[1] Fokkens WJ, Lund VJ, Hopkins C, Hellings PW, Kern R, Reitsma S, Toppila-Salmi S, Bernal-Sprekelsen M, Mullol J, Alobid I, Terezinha Anselmo-Lima W, Bachert C, Baroody F, von Buchwald C, Cervin A, Cohen N, Constantinidis J, De Gabory L, Desrosiers M, Diamant Z, Douglas RG, Gevaert PH, Hafner A, Harvey RJ, Joos GF, Kalogjera L, Knill A, Kocks JH, Landis BN, Limpens J, Lebeer S, Lourenco O, Meco C, Matricardi PM, O’Mahony L, Philpott CM, Ryan D, Schlosser R, Senior B, Smith TL, Teeling T, Tomazic PV, Wang DY, Wang D, Zhang L, Agius AM, Ahlstrom-Emanuelsson C, Alabri R, Albu S, Alhabash S, Aleksic A, Aloulah M, Al-Qudah M, Alsaleh S, Baban MA, Baudoin T, Balvers T, Battaglia P, Bedoya JD, Beule A, Bofares KM, Braverman I, Brozek-Madry E, Richard B, Callejas C, Carrie S, Caulley L, Chussi D, de Corso E, Coste A, El Hadi U, Elfarouk A, Eloy PH, Farrokhi S, Felisati G, Ferrari MD, Fishchuk R, Grayson W, Goncalves PM, Grdinic B, Grgic V, Hamizan AW, Heinichen JV, Husain S, Ping TI, Ivaska J, Jakimovska F, Jovancevic L, Kakande E, Kamel R, Karpischenko S, Kariyawasam HH, Kawauchi H, Kjeldsen A, Klimek L, Krzeski A, Kopacheva Barsova G, Kim SW, Lal D, Letort JJ, Lopatin A, Mahdjoubi A, Mesbahi A, Netkovski J, Nyenbue Tshipukane D, Obando-Valverde A, Okano M, Onerci M, Ong YK, Orlandi R, Otori N, Ouennoughy K, Ozkan M, Peric A, Plzak J, Prokopakis E, Prepageran N, Psaltis A, Pugin B, Raftopulos M, Rombaux P, Riechelmann H, Sahtout S, Sarafoleanu CC, Searyoh K, Rhee CS, Shi J, Shkoukani M, Shukuryan AK, Sicak M, Smyth D, Sindvongs K, Soklic Kosak T, Stjarne P, Sutikno B, Steinsvag S, Tantilipikorn P, Thanaviratananich S, Tran T, Urbancic J, Valiulius A, Vasquez de Aparicio C, Vicheva D, Virkkula PM, Vicente G, Voegels R, Wagenmann MM, Wardani RS, Welge-Lussen A, Witterick I, Wright E, Zabolotniy D, Zsolt B, Zwetsloot CP (2020) European Position Paper on Rhinosinusitis and Nasal Polyps 2020. Rhinology 58(Suppl S29), 1–464. 10.4193/Rhin20.600. PMID: 32077450. 32077450

[2] Orlandi RR, Kingdom TT, Smith TL, Bleier B, DeConde A, Luong AU, Poetker DM, Soler Z, Welch KC, Wise SK, Adappa N, Alt JA, Anselmo-Lima WT, Bachert C, Baroody FM, Batra PS, Bernal-Sprekelsen M, Beswick D, Bhattacharyya N, Chandra RK, Chang EH, Chiu A, Chowdhury N, Citardi MJ, Cohen NA, Conley DB, DelGaudio J, Desrosiers M, Douglas R, Eloy JA, Fokkens WJ, Gray ST, Gudis DA, Hamilos DL, Han JK, Harvey R, Hellings P, Holbrook EH, Hopkins C, Hwang P, Javer AR, Jiang RS, Kennedy D, Kern R, Laidlaw T, Lal D, Lane A, Lee HM, Lee JT, Levy JM, Lin SY, Lund V, McMains KC, Metson R, Mullol J, Naclerio R, Oakley G, Otori N, Palmer JN, Parikh SR, Passali D, Patel Z, Peters A, Philpott C, Psaltis AJ, Ramakrishnan VR, Ramanathan M Jr, Roh HJ, Rudmik L, Sacks R, Schlosser RJ, Sedaghat AR, Senior BA, Sindwani R, Smith K, Snidvongs K, Stewart M, Suh JD, Tan BK, Turner JH, van Drunen CM, Voegels R, Wang Y, Woodworth BA, Wormald PJ, Wright ED, Yan C, Zhang L, Zhou B (2021) International consensus statement on allergy and rhinology: Rhinosinusitis 2021. International Forum of Allergy & Rhinology 11, 213–739. 10.1002/alr.22741. 33236525

[3] Arcimowicz M (2024) Rational treatment of acute rhinosinusitis in the context of increasing antibiotic resistance. Otolaryngologia Polska 78, 1–11. 10.5604/01.3001.0054.7506. 39540274

[4] Perić A (2022) Acute rhinosinusitis – Pathogenesis, diagnosis and treatment. Galenika Medical Journal 1, 72–77. 10.5937/Galmed2201072P [Article in Serbian]

[5] Hoffmans R, Wagemakers A, van Drunen C, Hellings P, Fokkens W (2018) Acute and chronic rhinosinusitis and allergic rhinitis in relation to comorbidity, ethnicity, and environment. PLoS One 13, e0192330. 10.1371/journal.pone.0192330. 29401486 PMC5798836

[6] Sunyecz I, Hunt C, Ramadan HH, Makary CA (2024) Role of sinonasal anatomic variants in recurrent acute rhinosinusitis. Laryngoscope 134, 3489–3492. 10.1002/lary.31388. 38451036

[7] Salman FM, Dasgupta R, Eldeirawi KM, Nyenhuis SM, Lee VS (2023) Associations of community-level particulate matter with high-acuity visit presentation for sinusitis. American Journal of Otolaryngology 44, 103739. 10.1016/j.amjoto.2022.103739. 36580742 PMC10033369

[8] Günaydın RÖ, Eroğlu E, Tellioğlu B, Emiralioğlu N, Özçelik HU, Yalçın E, Doğru D, Kiper EN (2023) Evaluation of otorhinolaryngological manifestations in patients with primary ciliary dyskinesia. International Journal of Pediatric Otorhinolaryngology 168, 111520. 10.1016/j.ijporl.2023.111520. 36990030

[9] Jaume F, Valls-Mateus M, Mullol J (2020) Common cold and acute rhinosinusitis: Up-to-date management in 2020. Current Allergy and Asthma Reports 20, 28. 10.1007/s11882-020-00917-5. 32495003 PMC7266914

[10] Mullol J, Alobid I, Mariño-Sánchez F, Izquierdo-Domínguez A, Marin C, Klimek L, Wang DY, Liu Z (2020) The loss of smell and taste in the COVID-19 outbreak: A tale of many countries. Current Allergy and Asthma Reports 20, 61. 10.1007/s11882-020-00961-1. 32748211 PMC7397453

[11] Arcimowicz M (2021) Acute sinusitis in daily clinical practice. Otolaryngologia Polska 75, 40–50. 10.5604/01.3001.0015.2378. 34552023

[12] Eccles R (2011) Mechanisms of the symptoms of rhinosinusitis. Rhinology 49, 131–138. 21751530

[13] Perić A (2019) The Use of Herbal Drugs in Therapy of Acute and Chronic Rhinosinusitis. Belgrade, Serbia: Medija Centar Odbrana, pp. 141–154. [Textbook in Serbian]

[14] Kolodziej H (2011) Antimicrobial, antiviral, and immunomodulatory activity studies of *pelargonium sidoides* (EPs® 7630) in the context of health promotion. Pharmaceuticals (Basel) 4, 1295–1314. 10.3390/ph4101295. 27721327 PMC4060126

[15] Brendler T and van Wyk BE (2008) A historical, scientific, and commercial perspective on the medicinal use of *pelargonium sidoides* (*Geraniaceae*). Journal of Ethnopharmacology 119, 420–433. 10.1016/j.jep.2008.07.037. 18725280

[16] Bachert C, Schapowal A, Funk P, Kieser M (2009) Treatment of acute rhinosinusitis with the preparation from *pelargonium sidoides* EPs 7630: A randomized, double-blind, placebo-controlled trial. Rhinology 47, 51–58. 19382496

[17] Witte K, et al. (2015) The *pelargonium sidoides* extract EPs 7630 drives the innate immune defense by activating selected MAP kinase pathways in human monocytes. PLoS One 10, e0138075. 10.1371/journal.pone.0138075. 26406906 PMC4583277

[18] Witte K, Koch E, Volk HD, Wolk K, Sabat R (2020) The herbal extract EPs® 7630 increases the antimicrobial airway defense through monocyte-dependent induction of IL-22 in T cells. Journal of Molecular Medicine (Berlin, Germany) 98, 1493–1503. 10.1007/s00109-020-01970-3. 32948884 PMC7524690

[19] Chen Y, Geng Y, Jiang J, Xiong G, Lei C (2023) Smell and taste dysfunction in patients infected with the omicron variant of severe acute respiratory syndrome coronavirus-2. Acta Oto-Laryngologica 143, 489–494. 10.1080/00016489.2023.2223243. 37326433

[20] Ozer Ozturk E, Aslan M, Bayındır T (2022) The effect of COVID-19 on nasal mucociliary clearance. Acta Oto-Laryngologica 142, 329–332. 10.1080/00016489.2022.2048072. 35294841

[21] Papies J, Emanuel J, Heinemann N, Kulić Ž, Schroeder S, Tenner B, Lehner MD, Seifert G, Müller MA (2021) Antiviral and immunomodulatory effects of *pelargonium sidoides DC.* Root extract EPs® 7630 in SARS-CoV-2-infected human lung cells. Frontiers in Pharmacology 12, 757666. 10.3389/fphar.2021.757666. 34759825 PMC8573200

[22] Perić A, Vezmar Kovačević S, Barać A, Gaćeša D, Perić AV, Vojvodić D (2020) Effects of *pelargonium sidoides* extract on chemokine levels in nasal secretions of patients with non-purulent acute rhinosinusitis. Journal of Drug Assessment 9, 145–150. 10.1080/21556660.2020.1838176. 33209511 PMC7646548

[23] Thäle C, Kiderlen A, Kolodziej H (2008) Anti-infective mode of action of EPs 7630 at the molecular level. Planta Medica 74, 675–681. 10.1055/s-2008-1034324 18584813

[24] Jekabsone A, Sile I, Cochis A, Makrecka-Kuka M, Laucaityte G, Makarova E, Rimondini L, Bernotiene R, Raudone L, Vedlugaite E, Baniene R, Smalinskiene A, Savickiene N, Dambrova M (2019) Investigation of antibacterial and antiinflammatory activities of proanthocyanidins from *pelargonium sidoides* DC root extract. Nutrients 11, 2829. 10.3390/nu11112829. 31752295 PMC6893413

[25] Perić A, Gaćeša D, Barać A, Sotirović J, Perić AV (2020) Herbal drug EPs 7630 *versus* amoxicillin in patients with uncomplicated acute bacterial rhinosinusitis: A randomized, open-label study. The Annals of Otology, Rhinology, and Laryngology 129, 969–976. 10.1177/0003489420918266. 32456442

[26] Bachert C (2020) Evidence-based management of acute rhinosinusitis with herbal products. Clinical Phytoscience 6, 85. 10.1186/s40816-020-00231-7.

[27] Perić A, Vezmar Kovačević S, Barać A, Perić AV, Vojvodić D (2020) Effects of *pelargonium sidoides* extract vs roxithromycin on chemokine levels in nasal secretions of patients with uncomplicated acute rhinosinusitis. Laryngoscope Investig Otolaryngol 6, 25–33. 10.1002/lio2.514. PMC7883607 33614926

[28] da Silva MF, de Araújo-Júnior JX, da Silva-Júnior EF, Heimfarth L, Martins-Filho PR, Quintans JSS, Quintans-Júnior LJ (2022) Bradykinin-target therapies in SARS-CoV-2 infection: Current evidence and perspectives. Naunyn-Schmiedeberg’s Archives of Pharmacology 395, 275–283. 10.1007/s00210-022-02206-6. 35089406 PMC8795307

[29] van de Veerdonk FL, Kouijzer IJE, de Nooijer AH, van der Hoeven HG, Maas C, Netea MG, Brüggemann RJM (2020) Outcomes associated with use of a kinin B2 receptor antagonist among patients with COVID-19. JAMA Network Open 3, e2017708. 10.1001/jamanetworkopen.2020.17708. 32789513 PMC7426743

[30] Xavier-Santos JB, Passos JGR, Gomes JAS, Cruz JVC, Alves JSF, Garcia VB, da Silva RM, Lopes NP, Araujo-Junior RF, Zucolotto SM, Silva-Junior AA, Félix-Silva J, Fernandes-Pedrosa MF (2022) Topical gel containing phenolic-rich extract from Ipomoea pes-capre leaf (*Convolvulaceae*) has anti-inflammatory, wound healing, and antiophidic properties. Biomedicine & Pharmacotherapy 149, 112921. 10.1016/j.biopha.2022.112921. 36068780

